# There is more to nutrition and cardiovascular disease than ultra-processing— authors’ reply

**DOI:** 10.1016/j.lanepe.2024.101142

**Published:** 2024-12-11

**Authors:** Fernanda Rauber, Maria Laura da Costa Louzada, Kiara Chang, Carlos Augusto Monteiro, Eszter P. Vamos, Renata Bertazzi Levy

**Affiliations:** a Department of Preventive Medicine, School of Medicine, University of São Paulo, São Paulo 01246-903, Brazil; b Center for Epidemiological Research in Nutrition and Health, University of São Paulo, São Paulo 01246-904, Brazil; c Department of Nutrition, School of Public, University of São Paulo, São Paulo 01246-904, Brazil; d Public Health Policy Evaluation Unit, School of Public Health, Imperial College London, London W12 0BZ, United Kingdom

We would like to thank David Benton and Hayley Anne Young for their interest in our study^1^ and for providing feedback on our findings. We welcome the opportunity to address some of the concerns raised and to clarify certain aspects of our research.

First, we would like to clarify that our study did not investigate associations between macronutrients and cardiovascular disease (CVD), as stated in the letter. Instead, we examined CVD risk associated with the dietary contribution of food groups, considering both plant or animal origin and levels of food processing. Thus, the statement about non-linear relationships between macronutrients and CVD reported by this study is erroneous.

The letter also raises a question about sugar and its role in cardiovascular risk. We would like to clarify that the Nova classification system^2^ used in our study, distinguishes between sugar as a processed culinary ingredient (Nova group 2) and ultra-processed foods (Nova group 4), which often contain significant amounts of added sugars, industrial substances, and additives. Studies have consistently shown that as the dietary contribution of ultra-processed foods increases, so does the intake of added sugars, highlighting the key role that ultra-processed foods play in driving total sugar consumption.^3^

A central point raised by the authors of the correspondence is whether alternative approaches to analysing food should have been considered before concluding that we should avoid ultra-processed foods. They also ask why we focus on the manner of food production rather than the nature of the resulting food. To clarify, our emphasis on the processing level stems from a growing body of evidence that identifies the negative health impacts of ultra-processed foods,^4^ irrespective of their individual nutrient content.^5^ The Nova concept challenges the current nutrition paradigm that understands the healthfulness of foods based on the nutrient composition of the final product, often extracted from their dietary pattern context. The Nova classification explicitly considers the consequences of industrial processing itself, which often alters food structure, increases the presence of additives, and contributes to hyper-palatability, all of which can lead to overconsumption and poor health outcomes. For example, Hall et al.,^6^ in a controlled randomized crossover study, showed that even when nutrient profiles were matched, participants consuming an ultra-processed diet consumed, on average, 500 kcal more per day compared to those on an unprocessed diet. This indicates that factors beyond simple nutrient composition—such as the way involving ultra-processed foods are produced and their effects on satiety and eating behaviour—play a significant role in health outcomes. Additionally, Dicken and Batterham,^5^ in a review of cohort studies, found that “the majority of the associations between ultra-processed foods, obesity, and health-related outcomes remain significant and unchanged in magnitude after adjustment for diet quality or pattern.” These findings suggest that ultra-processed foods have negative health consequences that are independent of dietary quality or overall dietary patterns.

We fully agree with the authors that diet is a complex and integrated entity and different dietary approaches could be considered. However, the Nova classification was specifically developed to address this complexity by distinguishing between minimally processed and ultra-processed foods. Our study, designed around this classification, sought to reflect real-world eating behaviours and broader dietary patterns rather than focusing on individual foods in isolation.

In conclusion, while we appreciate the engagement with our study, we believe that some of the critiques stem from a misunderstanding of both the Nova classification and the scope of our research. A growing body of evidence increasingly points to the adverse health consequences of ultra-processed foods, not solely due to their nutrient content, but because of the way they are processed. We hope this response helps clarify these points and further contributes to the understanding the critical role food processing plays in public health.

## Contributors

F Rauber wrote the first draft and all authors reviewed and edited it.

## Declaration of interests

The authors declare no conflict of interest.
